# Compressibility of Natural Rubber at Pressures Below 500 kg/cm^2^

**DOI:** 10.6028/jres.068A.022

**Published:** 1964-06-01

**Authors:** Lawrence A. Wood, Gordon M. Martin

## Abstract

The specific volumes of unvulcanized natural rubber and of a peroxide-cured vulcanizate of natural rubber were measured at pressures of 1–500 kg/cm^2^ at temperatures from 0 to 25 °C. Observations on mercury-filled dilatometers were made through a window in the pressure system. No time effects or hysteresis phenomena were observed. The specific volume *V* in cm^3^/e over the range studied can be represented by

$$V=V_{0,25}\left\{1+A\left(t-25\right)\right\}\left\{1+\left[\alpha_{25}+k_{1}\left(t-25\right)\right]P+\left[\beta_{25}+k_{2}\left(t-25\right)\right]P^{2}\right\}$$

where *P* is the pressure in kg/cm^2^, and *t* the temperature in °C. The constants for the unvulcanized and for the peroxide-cured samples are:

*V*_0_,_25_= 1.0951 and 1.1032 cm^3^/g;
10^4^*A* = 6.54 and 6.36 per degree;
10^6^*α*_25_= −50.5 and −50.4 (kg/cm^2^)^−1^;
10^6^*k*_1_ = −0.227 and −0.203 per degree;
10^9^*β*_25_= 10 and 11.5 (kg/cm^2^)^−2^;
and 10^9^*k*_2_=0.048 and 0.073 per degree, respectively. The compressibility of unvulcanized natural rubber at 25° and 1 kg/cm^2^ is thus 50.5×10^−6^ (kg/cm^2^)^−1^ falling to 40.6×10^−6^ (kg/cm^2^) ^−1^ at a pressure of 500 kg/cm^2^. It is concluded that a low degree of vulcanization produces no significant changes in the constants listed. The values are not far different from those obtained by extrapolating to zero sulfur content the observations of Scott on the rubbersulfur system. Calculations of values of compressibility (and its reciprocal the bulk modulus), “internal pressure”, bulk wave velocity, difference between specific heats, and several other physical properties are in reasonable agreement with those obtained by direct observation by other workers. For the prediction of values at pressures above 500 kg/cm^2^ the use of the Tait equation is recommended.

## 1. Introduction

The compressibility (or its reciprocal the bulk modulus) is an important physical property of rubber, frequently required in practical applications and in calculations relating to mechanical properties. The quantity is also called the bulk compliance. When this is known, the change of volume on stretching can be calculated from observations of stress and strain, and compared with direct experimental observations [1] 1 or utilized in calculations of the relative contributions of energy and entropy in thermodynamic studies of rubber elasticity [2]. Other relations requiring values of compressibility are discussed in later sections of this paper.

Previous experimental measurements have seldom covered the proper ranges of variables to yield accurate values of the compressibility of unvulcanized or lightly vulcanized rubber near atmospheric pressure. Our paper presents data obtained on unvulcanized natural rubber and on a peroxide-cured natural rubber vulcanizate at pressures between 1 and 500 kg/cm^2^ at temperatures between 0 and 25 °C.

## 2. Experimental Arrangement

The unvulcanized sample was prepared from natural rubber latex by the purification procedure of McPherson [3]. An analysis showed 98.7 percent rubber hydrocarbon, 0.44 percent protein, and 0.18 percent ash. To this sample 1.00 percent of phenyl-beta-naphthylamine was added as an antioxidant. Another portion of this sample was used in crystallization studies by Roberts and Mandelkern [4], and Martin and Mandelkern [5]. The sample was made into a flat sheet 6×6×0.080 in. by molding at 100 °C.

The other sample was prepared from a lot of commercially purified rubber obtained from the United States Rubber Co., designated as No. 2103A, Batch 7505. To 100 parts of polymer 3 parts of dicumyl peroxide (“Dicup”) were added and the mixture cured 45 min at 140 °C.

The samples, which were relatively transparent, were examined carefully after molding or curing. No visible bubbles or other inhomogeneities were noted.

Specimens of each sample, weighing about 4 g each, were placed in mercury-filled volume dilatometers with 2 mm capillaries. The volume was determined following the practices developed in this laboratory some years ago [6].

The specific volume of a portion of each sample was determined at atmospheric pressure at 25° by hydrostatic weighings in water [7].

The mercury-filled dilatometers were completely immersed in an oil (Plexol) contained in a system [5] designed for the application of pressure at various temperatures. The pressure was built up by the operation of a piston actuated by a manually operated screw. It could be read on a precision Bourdontype gage. Observations of the level of the mercury in the dilatometer capillary were made through a tempered Pyrex glass window.

The height of the mercury in the capillary was read to the nearest 0.005 cm with a precision cathetometer, as the pressure was raised in increments of 100 kg/cm^2^ from 0 to 500 kg/cm^2^ gage pressure.

The values of compressibility found were so small that the change in volume produced by a change in pressure of 1 kg/cm^2^ was always less than 1 in the last digit reported. Thus no distinction needed to be drawn between gage pressure and absolute pressure, or between observations made at atmospheric pressure as compared with those which might have been conducted at zero pressure.

Once thermal equilibrium was established (within a period of about 15 min), no time effects or hysteresis phenomena were observed over the ranges of pressure and temperature investigated. We consider that our results are the low-frequency limits of the quantities displayed in the viscoelastic spectrum [8].

The total volume change observed was corrected for the volume change of the mercury and the glass, and combined with the specific volume determined at 1 atm at 25° to yield the values of the specific volume given in table 1. These are the complete basic data of this paper.

## 3. Calculations

It was found possible to represent the specific volume *V* over the range of observation by an equation of the following form:

$$V=V_{0,25}\left\{1+A\left(t-25\right)\right\}\left\{1+\left[\alpha_{25}+k_{1}\left(t-25\right)\right]P+\left[\beta_{25}+k_{2}\left(t-25\right)\right]P^{2}\right\}$$ (1)

where *P* is the pressure, *t* the temperature in °C, and *V*_0,25_, *A, α*_25_ *β*_25_, *k*_1_ and *k*_2_ are constants.

The compressibility *B* is defined here as

$$-\left(1/V_{0}\right)\left(\partial V/\partial P\right)$$

where *V*_0_ is the specific volume at zero pressure at any temperature. For materials conforming to eq (1) one finds:

$$V_{0}=V_{0,25}\left\{1+A\left(t-25\right)\right\}$$ (2)

and, by differentiation of eq (1),

$$B=-\left[\alpha_{25}+k_{1}\left(t-25\right)\right]-2\left[\beta_{25}+k_{2}\left(t-25\right)\right]P.$$ (3)

From this one obtains

$$\frac{\partial B}{\partial t}=-k_{1}-2k_{2}P$$ (4)

and

$$\frac{\partial B}{\partial P}=-2\left[\beta_{25}+k_{2}\left(t-25\right)\right].$$ (5)

The relations just given are simpler and calculations from them are easier when the compressibility is defined as −1/*V_0_*(∂*V/*∂*P*), in accordance with the usual practice [9], than when use is made of the *“* instantaneous” compressibility, defined as −(1*/V*) (∂*V/*∂*P*). The latter quantity, of course, is readily obtained from the former by multiplying it by *V*_0_/*V.* The difference between the two quantities is never greater than a few percent over the present range of investigation.

The constant *V*_0,25_ was determined, as already mentioned, by a direct measurement of the specific volume at atmospheric pressure and 25 °C by hydrostatic weighings. The constant *A* was determined by a least squares computation based on eq (2) relating specific volume and temperature for the observations at atmospheric pressure.

The remaining four constants in eq (1) were determined by least-squares computation based on eq (3). Here the mean compressibility over each range of 100 kg/cm^2^ was computed from the finite changes observed, and taken as the compressibility at the midpoint of the range. The bracketed quantities in eq (3) were determined at constant temperatures as the intercept and slope of the predicted linear relation between the compressibility *B* and the pressure *P.* The constants *α*_25_ and *k*_1_ were then determined by least squares as the intercept and slope in the predicted linear relation between the first bracketed quantity and the temperature. The constants *β*_25_ and *k*_2_ were determined similarly from the second bracketed term. A check on the values was obtained by conducting the computations in reverse order; that is, now the pressure was first considered constant as the temperature was varied and then the temperature taken as constant while the pressure was varied.

The values of the constants obtained in this manner were:
*Unvulcanized sample**Peroxide-cured sample**V*_0,25_1.09511.103210^4^
*A*6.54    6.36    10^6^
*α*_25_−50.5      −50.4      10^6^
*k*_1_−0.227  −0.203  10^9^
*β*_25_10.0      11.5      10^9^
*k*_2_0.048  0.073  The pressures are expressed in kg/cm^2^, the temperatures in °C, and the specific volumes in cm^3^/g.

The specific volumes calculated by eq (1) using these constants reproduce quite well the original data given in table 1. The differences between calculated and observed values are shown in table 2. The most serious deviations are in the values for the unvulcanized sample at 20°, where the calculated values are systematically too low. The values for the vulcanizate at 20° also tend to be too low, but the difference is not as great.

The lots of raw rubber used in preparing the two samples were of different origin and were purified by different methods. Consequently the specific volumes *V*_0,25_ of the unvulcanized materials differed. In the first case, *V*_0,25_ was 1.0951 cm^3^/g as shown in table 1; in the other, it was observed to be 1.1074 cm^3^/g. The addition of the dicumyl peroxide to the second sample gave a mixture with a specific volume about 1.1040 cm^3^/g, and on curing this fell to the value 1.1032 cm^3^/g shown in table 1.

The compressibility values calculated by eq (3), using the constants obtained above, are given in table 3. The values of (1/*V*_0,25_)(∂*V*/∂*t*), obtained by calculations based on a differentiation of eq (1), are given in table 4. The change in this quantity with changes in temperature is seen to be quite small, and will be regarded as negligible in this range.

## 4. Comparison With Similar Previous Direct Observations

The pressure ranges, temperatures, and samples used in six important previous compressibility studies on rubber are given in table 5. It will be noted that only three authors investigated pressures below 500 kg/cm^2^, only a few used pure-gum vulcanizates, and only one made measurements on unvulcanized rubber. Copeland’s experiments were adiabatic rather than isothermal.

The only cases yielding values which can reasonably be compared with the present results are the studies of Scott [11] and of Naunton (as calculated by McPherson) [13]. This comparison is made in table 6, where for uniformity all the pressures are to be measured in dynes/cm^2^ instead of kg/cm^2^ (1 kg/cm^2^=980665 dynes/cm^2^). This requires a change of exponent and an increase of 2 percent in the previously given coefficients related to the first power of the pressure, and of 4 percent in those related to the second power. These units will be used in the remainder of this paper. The values ascribed to Scott represent extrapolations to 0 and 2 percent sulfur respectively, his observations extending only over the range 3–31 percent sulfur. The vulcanizates used in the various studies were all somewhat different. Scott’s values refer to the rubber-sulfur system; the composition of Naunton’s “pure-gum vulcanizate” was not specified; and the present work was on a peroxide-cured rubber.

The agreement of our values with those published previously is regarded as very good, especially when one considers the differences in the composition of the vulcanizates and the fact that some of the constants are obtained as derivatives of values directly observed.

## 5. Calculations of Internal Pressure

The “internal pressure” *P_i_* of a liquid [16,17,18,19] is defined in thermodynamics as the change in internal energy *U* with change in volume.

$$P_{i}\equiv {\left(\frac{\partial U}{\partial V}\right)}_{T}=T{\left(\frac{\partial P}{\partial T}\right)}_{V}-P.$$ (6)

It will soon be evident from the numerical values below that for an applied pressure *P* of approximately 1 atm the second term is negligible compared with the first. The temperature in this instance must be expressed in °K. By differentiation of eq (2) one obtains:

$$A=\frac{1}{V_{0,25}}\frac{\partial V_{0}}{\partial T}.$$ (7)

Thus

$$\frac{A}{B}=\frac{V_{0}}{V_{0,25}}\frac{\partial P}{\partial T}$$ (8)

and

$$P_{i}=\frac{V_{0,25}}{V_{0}}\frac{A}{B}T.$$ (9)

The values calculated by eq (9) from the present data at different temperatures and a pressure of 1 atm are given in the fifth and sixth columns of table 7.

Allen, Gee, and coworkers [17] made a direct measurement of the quantity *T*∂*P/*∂*T* for a natural rubber vulcanízate containing 3.5 parts of sulfur and an accelerator. They obtained values of 88.6, 87.4, and 86.1 cal/cm^3^ at 20, 30, and 40 °C, respectively. The comparison with the corresponding figures in table 7 is exceedingly satisfactory. They report furthermore that the internal pressure of most polymers is approximately 20–40 percent larger than the cohesive energy density determined as the square of *δ*, the solubility parameter measured in swelling experiments. This relation is apparently valid for rubber also, since values of *δ*^2^ ranging from 64–70 cal/cm^3^ are normally reported for natural rubber vulcanizates [20, 21].

## 6. Calculations of Adiabatic Compressibility

The compressibility values were measured in the present work under isothermal conditions. For many applications, particularly where compressional waves are involved, the adiabatic compressibility is of interest. The difference between the isothermal compressibility *B* and the adiabatic compressibility *B_a_* has often been calculated from the relation [22]

$$B-B_{a}=A^{2}VT/C_{p}$$ (10)

where *A* is the thermal expansion coefficient, *V* the specific volume, *T* the temperature in °K, and *C_p_* the specific heat at constant pressure. Taking the values of *C_p_* (shown in table 7) from the work of Bekkedahl and Matheson [23] as the same for both unvulcanized and peroxide-cured rubber, and the other quantities from the present work, one obtains the values shown in the table for *A*^2^*VT/C_p_* and the corresponding adiabatic compressibility *B_a_*.

## 7. Comparison With Measurements Involving Bulk Wave Velocities

The adiabatic compressibility is the major factor determining the velocity of propagation of compressional waves in a sheet of rubber. The effective modulus for this type of wave is (*K_a_*+4/3 *G*) where *K_a_* is the adiabatic bulk modulus (the reciprocal of the adiabatic compressibility *B_a_*) and *G* is the shear modulus. The general equation is then

$$v^{2}=V\left(K_{a}+4/3\;G\right)$$ (11)

where *v* is the velocity of the compressional wave and *V* the specific volume of the medium. The present work on the vulcanizate yields *K_a_*=22,600× 10^6^ dynes/cm^2^ at 25 °C and 1 atm. Assuming the reasonable value of 4.3 kg/cm^2^ for *G* one finds 4/3 *G*=5.7 kg/cm^2^=5.6×10^6^ dynes/cm^2^. Therefore this term can be neglected in the remainder of the present discussion over the range of variables considered here.

The adiabatic bulk modulus *K_a_*, the velocity *v* of a compressional wave, calculated from eq (11), and the acoustic impedance, defined as *v/V*, the ratio of velocity to specific volume [24, 25], are each given in table 8 at different temperatures, as calculated from the data in table 7. The significance of the quantity $Vv^{\frac{1}{3}}$ in the last column will be discussed later.

With dynamic measurements the modulus is expected to be in general a function of the frequency. Ivey, Mrowca, and Guth [26] measured the time shift and amplitude change occurring when a sheet of pure-gum natural rubbber vulcanizate was inserted into the path of a pulsed compressional wave. From the observations they computed bulk wave velocity and bulk modulus.

A decrease in velocity was noted as the frequency was reduced from 10 Mc/s to 1 Mc/s. The magnitude of the decrease was about 5 percent at 0 and 20°, and about 2 percent at 40°. Further decreases were noted between 1 Mc/s and 100 Kc/s amounting about 2 percent at 0° and much less than 1 percent at 20 and 40°. No perceptible decreases were noted between 100 kc/s and 50 kc/s at 0° or any higher temperature. Heydemann [27] likewise could find no variation of bulk modulus with frequency over a range from 0.1 to 10,000 c/s at 20° for natural rubber samples of widely different compositions and moduli. At any given temperature the velocity thus decreases monotonically with decreasing frequency approaching asymptotically a low-frequency limit. We know of no experimental evidence for or reason to expect any further variation at lower frequencies than those mentioned.

It can be concluded from these results that dynamic measurements on pure-gum natural rubber vulcanizates at frequencies of 1 Mc/s and below, when conducted at 0° and above, will yield values of velocity not more than about 2 percent greater, and values of bulk modulus not more than about 4 percent greater, than the respective low-frequency limits. This important conclusion means that in this region in which occur almost all practical applications of rubber, the variation of bulk modulus with frequency can be neglected. The shear modulus, on the other hand, shows appreciable dispersion for several decades below 1 Mc/s at 0°, finally reaching a minimum change of 1–2 percent per decade at the lowest frequencies.

**Figure 1:**
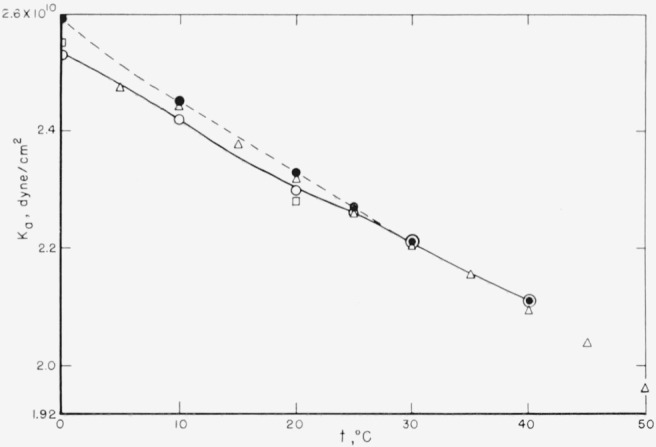
Adiabatic bulk modulus of rubber as a function of temperature. ● Present work—Unvulcanized ⊙ Present work—Peroxide-cured ⊡ Ivey et al. [26] figure 11—Pure-gum vulcanizate ◬ B. F. Goodrich data [28] on Rho C rubber vulcanizate

Figure 1 shows the adiabatic bulk modulus *K_a_* as a function of temperature as determined in the present work, in comparison with some values obtained by dynamic measurements reported in the literature.

The values ascribed to the B. F. Goodrich Co. [28] were calculated as *v*^2^/*V* from the sound velocity *v* at 5-degree temperature intervals, and a density value given as 0.98 g/cm^3^ at 25 °C. The rubber was a pure-gum natural rubber vulcanizate designated as Rho-C, Type 35000 (formerly 79SR32), with a Shore hardness of 35.

Values ascribed to Ivey et al. [26] are those read directly from the low-frequency limits of the bulk modulus in their figure 11 at 0 and 20°.

Other literature values at a single temperature (omitted from figure 1 for simplicity) are:
Temp. *°C**K_a_* *dyne/cm*^2^AuthorsReference  02.60×10^10^Cunningham and Ivey29    252.25Nolle and Mowry30    252.3Nolle31    302.22Cramer and Silver32    The agreement with the values shown in figure 1 at the respective temperatures is thoroughly satisfactory.

Cramer and Silver [32] measured the bulk modulus of a pure-gum vulcanízate at 30° by means of a resonant tube method [33] using a frequency of 1530 c/s.

It should be noted that the density of the normal pure-gum vulcanizates containing zinc oxide, sulfur, and an accelerator is approximately 0.98 g/cm^3^ (*V*=1.02 cm^3^/g), about 7 percent higher than that of the peroxide-cured vulcanízate used in the present work. According to the results just presented, this difference has no significant effect on the bulk modulus. The bulk wave velocities, however, are found to be about 3–4 percent lower than those given in table 8, in accordance with the predictions of eq (11).

In addition to the results just given, there are also literature values which are not properly comparable or do not agree so well with those we are reporting. For example, Wada and collaborators [34] made measurements on unvulcanized natural rubber latex, diluted with water until the rubber was about 10 percent by volume. They measured the velocity of a pulsed compressional wave in the suspension and compared it with that in the suspending liquid. The frequency used was 1 Mc/s. We see no obvious explanation for the fact that the bulk moduli they reported are about 10 percent lower than those we observed for unvulcanized rubber.

Heydemann [27] has reported values of dynamic compressibility for a number of rubber vulcanizates of unspecified composition. The highest value, which might be presumed to be that characteristic of a pure-gum vulcanizate, was 45.0×10^−12^ cm^2^/dyne at 20° corresponding to a bulk modulus of 2.22×10^10^ dyne/cm^2^. This is about 4 percent less than the values shown in figure 1. The discrepancy is not surprising in view in the uncertainty regarding the composition.

McKinney, Belcher, and Marvin [35] measured the dynamic compressibility of a vulcanizate containing 12.1 percent sulfur. Their values have not been tabulated, since their vulcanízate can not be regarded as a soft or lightly vulcanized rubber. It was in fact chosen to permit measurements in the vicinity of the glass transition. They found that the value of isothermal compressibility *B*, at 25° and 1 atm was 41.4×10^−12^ cm^2^/dyne. Scott [11] reports 37.5×10^−12^ cm^2^/dyne for a vulcanizate containing 12 percent sulfur.

Some observers have reported their results only in terms of the bulk wave velocity without giving density values. In many instances the densities of the pure-gum vulcanizates would be expected to be near 0.98 g/cm^3^, and the velocities 3–4 percent lower than those given in table 8, as already mentioned.

Unpublished measurements of Barnes [36] are reported as yielding a compressional wave velocity of about 1600 m/s, at 10,000 to 50,000 c/s, in essential agreement with the values in table 8.

The values of bulk wave velocity for natural rubber observed by Natta and Baccaredda [37] are also in close conformity with the extrapolation of the present values to higher temperatures. Assuming a value of ∂*v/*∂*t*=3.0 m/sec^−1^ deg^−1^ on the basis of the data in table 8, one obtains a velocity of about 1440 m/s at 70 °C for both unvulcanized and peroxide-cured rubbers. This is in good agreement with the values 1465 ± 20 m/s at this temperature observed directly by Natta and Baccaredda.

On the other hand, compressional wave velocities about 9 percent lower than those given in table 8 were measured by Levi and Philipp [38] who observed the frequencies at which successive maxima in transmission occurred in sheets about 1 mm thick, immersed in mercury. A dozen or more resonances were observed at frequencies between 1.5 and 8.6 Mc/s. The temperature was not specified. The velocities measured were 1430 and 1479 m/s for two different samples of unvulcanized evaporated latex and 1450 m/s for a pure-gum vulcanizate made from the first latex sample. A commercial soft-rubber vulcanizate gave 1440 m/s. No significant variation with frequency could be observed over the range studied.

The acoustic impedance *“ρc”* given in the next to the last column of table 8 governs the extent of reflection of compressional waves at the boundary of a medium. Zero reflection is obtained when the acoustic impedances on the two sides of the boundary are equal. This is important in underwater sound devices, where acoustically transparent protective materials are required for sonar domes, transducer covers, hydrophone tubes, and similar applications.

The acoustic impedance of fresh water [39, 40] increases from about 1403×10^−2^ CGS units at 0° to about 1493×10^−2^ at 25°, while that of sea water [41] rises from about 1490 to 1570×10^−2^ over the same range. In the region of greatest practical importance, 10–15 °C, the value for sea water is about 1530×10^−2^. As can be seen from the table, the acoustic impedance of rubber decreases with increasing temperature. Consequently a matching of *ρc* for sea water with that for rubber can occur at only a single temperature. From the values for rubber given in table 8 it is concluded that only slight increases in the quantity *ρc* are required to attain the value of 1530×10^−2^ at 10–15°. Experience verifies this conclusion and at least one commercial vulcanízate has been designed for this purpose [28]. At each temperature the density is higher, the velocity lower, and *ρc* slightly higher than the values used or given in table 8 for the peroxide-cured rubber.

Cramer and Silver [32] found that when an EPC (Easy Processing Channel) carbon black was used as a filler in natural rubber, there was a considerable increase in bulk modulus and a considerable increase in density. The velocity, depending as it does on the ratio of these quantities, was found to decrease slightly with filler content. The increase in density overbalanced the slight decrease in velocity so that the product *“ρc”* increased with increasing filler content. For rubber with a filler content sufficient to raise the density to 1.10 g/cm^3^ and the Shore hardness to 52, the Goodrich company [28] has reported similar results, except that they found at most temperatures a very slight increase in velocity as compared with the pure-gum vulcanizate.

## 8. Calculation of Values of Other Properties

The compressibility *B* may be combined with Young’s modulus *E* to give the value of Poisson’s ratio *μ.* From classical mechanics the general relation for an infinitesimal deformation is:

$$\mu =\frac{1}{2}-\frac{1}{6}BE.$$ (12)

For the peroxide-cured vulcanizate at 25° *B*_25_= 50.4×10^−12^ cm^2^/dyne (table 3) and a reasonable value of *E* would be 13 kg/cm^2^, yielding 0.49989 for Poisson’s ratio for an infinitesimal deformation.

The ratio *E/G* from classical mechanics is given by

$$E/G=3-\frac{1}{3}BE.$$ (13)

With the quantities already mentioned, this ratio is calculated as 2.99977 for the rubber vulcanizate.

The quantities measured in the present work may be utilized to compute the difference between the specific heats at constant pressure *C_P_* and at constant volume *C_V_*. The relation given by thermodynamic considerations [42] is

$$C_{P}-C_{V}=A^{2}VT/B.$$ (14)

The values given in table 7 for the unvulcanized rubber at 25 °C and at atmospheric pressure can be inserted into the right-hand member of the equation to yield 0.0648 cal/g °C for the difference in specific heats. This may be compared with a value of 0.066 cal/g °C at 27° given by Rehner [43] as the difference between *C_P_*= 1.891 j/g °C=0.452 cal/g °C measured directly by Bekkedahl and Matheson [23] and *C_v_*=0.386 cal/g °C calculated from the structure by a method described by Tarassov [44].

The ratio *C_P_/C_V_* can be obtained by combining equations (10) and (14).

$$C_{P}/C_{V}=B/B_{a}.$$ (15)

Values for *B/B_a_* computed from the data given in table 7 decrease from 1.184 at 0° to 1.160 at 40° for the unvulcanized material and from 1.170 at 0° to 1.152 at 40° for the peroxide-cured rubber. Independent values for *C_P_/C_V_* other than those derived from the values of *C_P_* and *C_V_* given in the preceding paragraph seem to be unavailable. The ratio in that instance is 1.171 at 27°.

The present results may be used to study the validity (for rubber) of the relation

$$MVv^{1/3}=R$$ (16)

where *M* is the molecular weight, *V* the specific volume, *v* the bulk wave velocity, and *R* is a constant, independent of temperature. This equation was proposed by Rao [45] some years ago as an empirical relation valid for organic liquids of low molecular weight.

Table 8 (col 5) shows that this relation holds reasonably well for the two rubbers over the range of temperature investigated, the molecular weight being assumed constant in eacli instance.

Lagemann and Corry [46] have shown how the value of *R* in Rao’s relation may be predicted (for compounds of low molecular weight) from structural considerations, utilizing the additivity of “bond increments”. In each mer unit of rubber (C_5_H_8_), for example, 8 C—H bonds would contribute 95.2 units each, 4 C—C bonds 4.25 each, and 1 C=C bond 129 units, for a total of 908. The value of *R* for the average molecule would then be 908 *n* and the molecular weight *M* would be 68.119 *n*, where *n* is an average degree of polymerization. Consequently the use of Lagemann and Cory’s bond increments would predict a value of 908/68.119 = 13.33 for *R/M*, the quantity given in table 8, col 5. The difference is about 4 percent.

Replacing *v* in Rao’s eq (16) by the value given by eq (11) (*G* being assumed negligible in comparison with *K_a_*), and differentiating with respect to *T* one obtains [47] at *P*=0:

$$\frac{\frac{1}{B_{a}}\frac{\partial B_{a}}{\partial T}}{\frac{1}{V}\frac{\partial V}{\partial T}}=7.$$ (17)

The left-hand member of this equation, as calculated from the numerical values at *P*=0 obtained in the present work, decreases from 8.3 at 0° to 7.2 at 40° for the unvulcanized rubber and from 7.4 at 0° to 6.6 at 40° for the peroxide-cured material.

The constants *k*_1_ and *A* are, respectively, the dominant factors in determining the numerator and denominator of the left-hand member of eq (17). The ratio of their values as measured here, consequently, is approximately that derived using Rao’s relation.

## 9. Values Predicted at Pressures Above 500 kg/cm^2^

It is not intended that eq (1) through (5) be used for pressures above 500 kg/cm^2^. It is well known from previous work that an expression involving higher powers of *P* is required instead of eq (1) for an adequate representation of the data.

For this purpose, the applicability of a relation known as the Tait equation has become increasingly apparent in recent years.^2^ The validity of this form of relation in representing data on hydrocarbons with molecular weights as high as 350 has been demonstrated by Cutler, McMickle, Webb, and Schiessler [48]. These authors give references to several previous applications of the relation but it seems not to have received the attention it deserves. For example, it is not mentioned in the well-known treatise of Bridgman [9].

The Tait equation can be written:

$$\frac{V_{0}-V}{V_{0}}=c\log_{10}\left(1+P/b\right)$$ (18)

where *b* and *c* are constants.

A change to natural logarithms and a series expansion yield:

$$\frac{V_{0}-V}{V_{0}}=0.4343\;c\left[\left(P/b\right)-\frac{1}{2}{\left(P/b\right)}^{2}+\frac{1}{3}{\left(P/b\right)}^{3}-\ldots \right].$$ (19)

From eq (1) if *α= α*_25_*+k*_1_(*t*−25) and *β*= *β*_25_+ *k*_2_(*t*−25), one obtains

$$\frac{V_{0}-V}{V_{0}}=\alpha P-\beta P^{2}.$$ (20)

It is apparent that eq (20) and its parent eq (1) represent only an approximation obtained by neglecting higher powers of pressure in eq (19). It is found on direct test that by the proper choice of constants *b* and *c* the Tait equation will yield as good agreement with observed values of volume as has already been shown in table 2 for the approximation. In other words, within the precision of our measurements the two equations are indistinguishable over the range of pressures below 500 kg/cm^2^. At 25° our data for the unvulcanized rubber were well represented by *c*=0.240 and *b* = 2050 kg/cm^2^ in the Tait equation; for the peroxide-cured rubber the corresponding values were *c* = 0.193 and *b* = 1640 kg/cm^2^.

Equation (20) was used in the present work below 500 kg/cm^2^ because it is simpler than eq (18), and the coefficients can be evaluated more readily. In view of the excellent agreement of calculated and observed values shown in table 2, it was not considered necessary to use the complete expansion. In each equation, of course, the variation of the coefficients with temperature can be calculated from observations at different temperatures. Previous work [48] on nonpolymeric compounds has found *c* to be independent of temperature and to have a value near 0.2.

The success of the Tait equation in representing data for hydrocarbon liquids up to the highest pressures investigated suggested an examination of its applicability to rubber at pressures above 500 kg/cm^2^. It was found that the data of Scott [11] (extending to about 800 kg/cm^2^), Naunton [13] (extending to about 1750 kg/cm^2^), Adams and Gibson [10] (extending to about 12,000 kg/cm^2^), and Bridgman [12] (extending to 25,000 kg/cm^2^) could all be well represented by expressions in the form of eq (18), with values of *b* in the range 15002200 kg/cm^2^ and values of *c* in the neighborhood of 0.2. None of these authors had utilized the Tait equation.

In view of the results just mentioned, the Tait equation is suitable for representing the volume of rubber at all pressures up to the limit of observation. It is to be strongly preferred for the range above 500 kg/cm^2^, where McPherson [13], for example, found it necessary to add a term involving *P*^3^ to eq (1).

The compressibility, obtained by differentiation of eq (18), is

$$B=\frac{0.4343\;c}{P+b}.$$ (21)

A series expansion of this expression in powers of *P* shows that eq (3) also is an approximation valid only for low pressures.

## 10. Conclusions

The compressibility values and their coefficients describing changes with pressure and temperature are not far different from those estimated by extrapolation from Scott's data on the rubber-sulfur system. There is no significant change of compressibility on vulcanization with dicumyl peroxide, and the changes in the coefficients are slight.

The compressibility at 25° extrapolated to zero pressure (*α*_25_) found here is slightly greater than that of cesium, the most compressible crystalline solid for which we have found values in the literature [49]. It is appreciably less than that of most normal organic liquids of low molecular weight. As already mentioned it is nearly the same as that of water, a distinctly abnormal liquid. This fact, while not of any obvious theoretical or structural significance, is of considerable practical importance since it becomes possible to make rubber vulcanizates which will have a minimum reflection or scattering of underwater sound waves, as already mentioned.

The value of *α*_25_ found here is essentially the same as that found by Jessup [50] to represent the compressibility of high viscosity petroleum oils (gas oils and lubricating oils) of the same specific volume as the rubber. This may be of theoretical significance since the structures involved may well be similar in all respects except molecular weight. Values given in the literature [51] for other organic oils (such as almond, castor, linseed, olive, and rapeseed oils) are nearly the same. Glycerine is apparently the only organic liquid appreciably less compressible than these.

Most of the limited number of available literature values for other elastomers are not far different from those for natural rubber. The values for semi-crystalline polymers and polymers below their glass transition temperatures tend to be somewhat lower.

The thermal expansion coefficient *A* is also found to have essentially the same value as that given for the high viscosity petroleum oils [50].

From the compressibility values and their coefficients it has been shown that one may calculate values for a considerable number of other properties of rubber, obtaining good agreement with available direct experimental values. These include internal pressures, sound wave velocities, and specific heats.

## Figures and Tables

**Table 1:** Observations of specific volume

Unvulcanized rubber				Peroxide-cured vulcanizate			
	
*P*	*V*_25_	*V*_20_	*V*_0_	*V*_25_	*V*_20_	*V*_10_	*V*_0_
							
*kg/cm*^2^	*cm*^3^*/q*	*cm*^3^*/g*	*cm*^3^*/g*	*cm*^3^*/g*	*cm*^3^*/g*	*cm*^3^*/g*	*cm*^3^*/g*
0	1.0951	1.0917	1.0772	1.1032	1.0998	1.0927	1.0857
100	1.0896	1.0864	1.0725	1.0977	1.0944	1.0876	1.0807
200	1.0845	1.0814	1.0679	1.0926	1.0894	1.0828	1.0763
300	1.0795	1.0764	1.0636	1.0877	1.0846	1.0782	1.0719
400	1.0748	1.0718	1.0593	1.0830	1.0801	1.0739	1.0678
500	1.0702	………	1.0554	1.0786	1.0756	1.0697	1.0637

**Table 2:** Difference between calculated and observed values of sveciiic volume

Unvulcanized rubber				Peroxide-cured vulcanizate			
	
*P* *kg/cm*^2^	*V*_25_ *cm^2^/g* ×10^−4^	*V*_20_ *cm*^3^*/g* ×*10*^−4^	*V*_0_ *cm*^3^*/g* ×*10*^−4^	*V*_25_ *cm*^2^*/g* ×*10*^−4^	*V*_20_ *cm/*^3^*g* ×*10*^−4^	*V*_10_ *cm*^3^*/g* ×*10*^−4^	*V*_0_ *cm*^3^*/g* ×*10*^−4^
							
0	0	−2	0	0	−1	0	0
100	1	−2	0	1	0	0	+1
200	0	−2	0	0	−1	0	0
300	0	−1	0	0	−1	0	0
400	−1	−1	+1	0	−2	−1	−1
500	0	……	0	0	0	−1	0

**Table 3:** Compressibility (−1/V_0_) (∂V/∂P)

Unvulcanized rubber				Peroxide-cured vulcanizate			
	
*kg/cm^2^*	*B*_25_	*B*_20_	*B*_0_	*B*_25_	*B*_20_	*B*_10_	*B*_0_
						
	*cm*^2^/*kg* ×*10*^−6^	*cm*^2^/*kg* ×*10*^−6^	*cm*^2^/*kg* ×*10*^−6^	*cm*^2^/*kg* ×*10*^−6^	*cm*^2^/*kg* ×*10*^−6^	*cm*^2^/*kg* ×*10*^−6^	*cm*^2^/*kg*×*10*^−6^
							
0	50.5	49.4	44.9	50.4	49.4	47.4	45.3
100	48.5	47.4	43.0	48.1	47.2	45.3	43.4
200	46.5	45.5	41.3	45.8	44.9	43.2	41.4
300	44.5	43.5	39.5	43.5	42.7	41.2	39.5
400	42.5	41.6	37.8	41.2	40.5	39.1	37.6
500	40.6	39.7	36.1	39.0	38.4	37.1	35.8

**Table 4:** Thermal expansivity (1/V_0,25_) (∂V/∂T)

Unvulcanized rubber				Peroxide-cured vulcanizate			
	
*kg/cm*^2^	Temperature			Temperature			
	
	25° deg^−1^ ×*10*^−4^	20° deg^−1^ ×*10*^−4^	0° deg^−1^ ×*10*^−4^	25° deg^−1^ ×*10*^−4^	20° deg^−1^ ×*10*^−4^	10° deg^−1^ ×*10*^−4^	0° deg^−1^ ×*10*^−4^
							
0	6.54	6.54	6.54	6.36	6.36	6.36	6.36
100	6.29	6.28	6.28	6.13	6.13	6.13	6.13
200	6.04	6.04	6.03	5.92	5.92	5.91	5.91
300	5.81	5.81	5.79	5.73	5.72	5.72	5.71
400	5.59	5.58	5.56	5.55	5.54	5.53	5.53
500	5.37	5.36	5.33	5.39	5.38	5.37	5.36

**Table 5:** Summary of six previous investidations

Authors	Ref	Year	Temp	Pressure range	Samples
					
			*°C*		
Adams & Gibson.	[10]	1930	25	1,000–12,000 bars.	1. Vulcanizate Rubber 90-Sulfur 10 2. Vulcanizate Rubber 90-Sulfur 4 Zinc Oxide Accel. 0.25
Scott	[11]	1935	10–85	1–800 bars	Vulcanizates: Rubber 97–69 Sulfur 3–31
Bridgman	[12]	1944	…………	2,000–25,000 bars.	Pure-gum vulcanizate
Naunton, cal culated by McPherson 1958.	[13]	1945	…………	48.5–1715 bars	Unvulcanized rubber Pure-gum vulcanizate
Copeland	[14]	1948	…………	1–345 bars.	1. Vulcanizate Rubber 100-Sulfur 8 2. Vulcanizate Rubber 100-Sulfur 2.5 Zinc Oxide Accel. 1
Weir	[15]	1953	10–81.5	1,000–10,000 bars.	Vulcanizates: Rubber 90–72 Sulfur 10–28

**Table 6:** Comparison of values of constants (Pressures measured in dynes/cm^2^)

Constant	Unvulcanized			Vulcanizates		
	
	Scott 0% S	Naunton	Present work	Scott 2% S	Naunton “pure-gum”	Present work, peroxide
						
*V*_0,25_	1.1015	…………	1.0951	1.0829	…………	1.1032
10^4^ *A*	6.63	…………	6.54	6.61	…………	6.36
10^12^ *α*_25_	−53.7	−53.8	−51.5	−51.0	−50.7	−51.4
10^12^ *k*_1_	−0.258	…………	−0.231	−0.262	…………	−0.207
10^21^ *β*_25_	12.0	13.6	10.4	11.3	11.8	12.0
10^21^ *k*_2_	0.083	…………	0.050	0.083	…………	0.076

**Table 7:** Values of thermodynamic quantities at 10^6^ dynes/cm^2^

*Unvulcanized rubber A*=6.54×10^−4^ (*deg C*)^−1^								

*T*		*V*	*B*	(*V*_0,25_/*V*_0_)(TA/B)		*C_p_*^b^	*A*^2^*VT/C_p_*	*B_a_*
						
*°C*	*°K*	*cm*^3^*/g*	*cm*^3^*/dyne*	*dyne/cm*^2^	*cal/cm*^3^	*j/g °C*	*cm*^2^*/dyne*	*cm*^2^/*dyne*
0	273.2	1.0072	45.7×10^−12^	3980×10^6^	95.0	1.785	7.05×10^−12^	38.6×10^−12^
10	283.2	1.0844	48.0	3900	93.2	1.816	7.23	40.8
20	293.2	1.0917	50.03	3820	91.4	1.856	7.38	42.9
25	298.2	1.0951	51.5	3790	90.5	1.881	7.43	44.1
30^a^	303.2	1.0987	52.7	3750	89.6	1.906	7.48	45.2
40^a^	313.2	1.1058	55.0	3680	88.1	1.956	7.57	47.4

*Peroxide-cured rubber A*=6.36×10^−4^ (*deg C*)^−1^								

0	273.2	1.0857	46.2	3820	91.3	1.785	6.71	39.5
10	283.2	1.0927	48.3	3780	90.2	1.816	6.89	41.4
20	293.2	1.0998	50.4	3710	88.7	1.856	7.03	43.4
25	298.2	1.1032	51.4	3690	88.2	1.881	7.08	44.3
30^a^	303.2	1.1067	52.4	3670	87.7	1.906	7.12	45.3
40^b^	313.2	1.1137	54.5	3620	86.6	1.956	7.21	47.3

a Values given at 30 and 40 °C for *V* and quantities derived from *V* are based on an extrapolation of eq (1).

b Values observed for unvulcanized rubber by Bekkedahl and Matheson [23].

**Table 8:** Bulk moduli, bulk wave velocities, and derived quantities at 10^6^ dynes/cm^2^

Unvulcanized rubber				

*t*	*K_a_*	*v*	*“ρc” =v/V*	$Vv^{\frac{1}{3}}\left(=R/M\right)$
				
°C	*dynes/cm*^2^	*m/sec*	*g cm*^−2^ *sec*^−^*^1^*	(*cm*^3^ *g*^−1^) (*m sec*^−1^)^1/3^
0	2.59×10^−10^	1670	1550×10^−2^	12.78
10	2.45	1630	1500	12.76
20	2.33	1600	1460	12.77
25	2.27	1580	1440	12.76
30^a^	2.21	1560	1420	12.74
40^a^	2.11	1530	1380	12.74

Peroxide-cured rubber				

0	2.53	1660	1530	12.85
10	2.42	1630	1490	12.86
20	2.30	1590	1450	12.83
25	2.26	1580	1430	12.85
30^a^	2.21	1560	1410	12.84
40^a^	2.11	1530	1380	12.83

a Values given at 30 and 40 °C are based on an extrapolation of eq (1).

## Footnotes

1 Figures in brackets indicate the literature references at the end of this paper.

2 We thank Professor Robert Simha of the University of Southern California for bringing the Tait equation to our attention.
